# Metabolic Syndrome: Is It Time to Add the Central Nervous System?

**DOI:** 10.3390/nu13072254

**Published:** 2021-06-30

**Authors:** Milagros Rojas, Mervin Chávez-Castillo, Daniela Pirela, Heliana Parra, Manuel Nava, Maricarmen Chacín, Lissé Angarita, Roberto Añez, Juan Salazar, Rina Ortiz, Samuel Durán Agüero, Marbel Gravini-Donado, Valmore Bermúdez, Edgar Díaz-Camargo

**Affiliations:** 1 Endocrine and Metabolic Diseases Research Center, School of Medicine, University of Zulia, Maracaibo 4004, Venezuela; migarocafi@gmail.com (M.R.); pirelacdaniela@gmail.com (D.P.); helianapp20@hotmail.com (H.P.); manuelnava_14@hotmail.com (M.N.); juanjsv18@hotmail.com (J.S.); 2 Psychiatric Hospital of Maracaibo, Maracaibo 4004, Venezuela; mervinch@hotmail.com; 3 Facultad de Ciencias de la Salud, Universidad Simón Bolívar, Barranquilla 08002, Colombia; m.chacin@unisimonbolivar.edu.co; 4 Escuela de Nutrición y Dietética, Facultad de Medicina, Universidad Andrés Bello, Sede Concepción 4260000, Chile; lisse.angarita@unab.cl; 5 Departamento de Endocrinología y Nutrición, Hospital General Universitario Gregorio Marañón, 28007 Madrid, Spain; robertojose.anez@salud.madrid.org; 6 Posgrado, Carrera de Medicina, Universidad Católica de Cuenca, Cantón de Cuenca 010101, Ecuador; rortiz@ucacue.edu.ec; 7 Facultad de Ciencias Para el Cuidado de la Salud, Universidad San Sebastián, Los Leones 8420524, Chile; samuel.duran@uss.cl; 8 Facultad de Ciencias Jurídicas y Sociales, Universidad Simón Bolívar, Barranquilla 080002, Colombia; mgravini1@unisimonbolivar.edu.co; 9 Facultad de Ciencias Jurídicas y Sociales, Universidad Simón Bolívar, Cúcuta 540006, Colombia; v.bermudez@unisimonbolivar.edu.co

**Keywords:** metabolic syndrome, insulin resistance, diabetes mellitus type 2, mild cognitive impairment, Alzheimer’s disease

## Abstract

Metabolic syndrome (MS) is a set of cardio-metabolic risk factors that includes central obesity, hyperglycemia, hypertension, and dyslipidemias. The syndrome affects 25% of adults worldwide. The definition of MS has evolved over the last 80 years, with various classification systems and criteria, whose limitations and benefits are currently the subject of some controversy. Likewise, hypotheses regarding the etiology of MS add more confusion from clinical and epidemiological points of view. The leading suggestion for the pathophysiology of MS is insulin resistance (IR). IR can affect multiple tissues and organs, from the classic “triumvirate” (myocyte, adipocyte, and hepatocyte) to possible effects on organs considered more recently, such as the central nervous system (CNS). Mild cognitive impairment (MCI) and Alzheimer’s disease (AD) may be clinical expressions of CNS involvement. However, the association between MCI and MS is not understood. The bidirectional relationship that seems to exist between these factors raises the questions of which phenomenon occurs first and whether MCI can be a precursor of MS. This review explores shared pathophysiological mechanisms between MCI and MS and establishes a hypothesis of a possible MCI role in the development of IR and the appearance of MS.

## 1. Introduction

Metabolic syndrome (MS) is a serious public health problem. It affects about 25% of the general population and, more alarmingly, around 40% of adults over 40 years old worldwide [1,2]. The definition of this syndrome has recently evolved to include a group of at least three of five cardio-metabolic abnormalities. These conditions include high blood pressure, central obesity, insulin resistance (IR), elevated blood triglycerides, and atherogenic dyslipidemia [3], which together lead to an increased risk of cardio-metabolic pathologies [4,5,6], as well as other diseases, such as arthritis [7] and some types of cancer [8].

Likewise, the presence of MS-related disorders also plays a role in the pathophysiology of neurological disorders [9], reflecting the association between deficiencies in secretion and action of insulin and mild cognitive impairment (MCI) [10]. MCI is defined as cognitive dysfunction that exceeds what is typically expected for age and educational level but does not meet the criteria for a major neurocognitive disorder. General functionality is preserved in MCI [11] and it can be best described as an intermediate state between cognitive impairment characteristic of aging and major neurocognitive conditions, such as Alzheimer’s disease (AD) [12,13].

Pathophysiological mechanisms of MCI have not been fully clarified. Thus, different hypotheses are proposed to explain the MCI/MS association [14]. A cyclical relationship seems to exist between IR and cognitive impairment, and the question of which phenomenon occurs first arises [15]. Additionally, if cognitive impairment precedes IR, it becomes a risk factor for developing MS. Therefore, this review briefly describes the history of MS and discusses clinical and preclinical findings that support the role of MS and IR as elements of pathophysiological mechanisms of cognitive impairment.

While most reviews on the topic focus on the MS-to-MCI relationship, this review goes beyond this by looking at the inverse relationship, examining available evidence regarding a new hypothesis that suggests that cognitive impairment could have a role in the development of IR and the appearance of MS. Among the mechanisms to be highlighted in this regard, the hyperphosphorylation of tau proteins and the formation of amyloid β (Aβ) plaques are proposed as alterations that go beyond the pathophysiology of Alzheimer’s disease (AD), and their role in the pathophysiology of insulin alterations is examined.

## 2. Metabolic Syndrome: Historical Aspects

The first studies of MS started almost 100 years ago when Eskil Kylin, in 1921, and Gregorio Marañón, in 1922, independently published in the same journal (Zentralblatt für Innere Medizin) papers with the same title, “diabetes mellitus and hypertension” [16,17]. Yet, not until 1981 did Hanefeld and Leonhardt use the term “metabolic syndrome” for the first time [18].

In 1988, Gerald Reaven hypothesized that IR was a common etiological factor for a group of disorders he termed “Syndrome X” [19,20]. He used this name to emphasize its unknown origin. At this time, the fundamental pathophysiological role of IR was known. This mechanism had been studied by researchers, such as Randle [21]. In subsequent years, DeFronzo, Ferrannini, and others used the term “Insulin Resistance Syndrome”, proposing that available evidence suggested its presence was the cause of MS [22].

The cause of MS and its components have been debated worldwide since the end of the 20th century. Many organizations, such as the World Health Organization (WHO) [23], the European Group for the Study of Insulin Resistance (EGIR) [24], the Adult Treatment Panel III (ATP-III) [25], the American Association of Clinical Endocrinologists (ACE/AACE) [26], and the International Diabetes Federation (IDF) [27], have proposed evolving diagnostic criteria. Some criteria have been progressively discarded and replaced with criteria that can be easily applied in daily clinical practice.

Finally, the IDF, the National Heart, Lung, and Blood Institute, the American Heart Association, the World Heart Federation, the International Atherosclerosis Society, and the International Association for the Study of Obesity made a joint statement in 2009 that concluded that a diagnosis of MS requires the presence of three or more of the following criteria: high abdominal circumference as defined for each geographical region, triacylglycerides (TAG) greater than or equal to 150 mg/dL, HDL levels less than 50 mg% in women or less than 40 mg% in men, systolic blood pressure (SBP) greater than or equal to 130 mmHg or diastolic blood pressure (DBP) greater than or equal to 85 mmHg, and glycemic levels greater than 100 mg/dL [3]. We now consider that the evolution of diagnostic criteria has reached a maturity level that makes it difficult to incorporate new criteria that are both easily recognized and provide useful clinical information [28]. IR continues to be the most widely accepted hypothesis to describe MS pathophysiology that involves various organs and associations with numerous diseases [29].

## 3. Mild Cognitive Impairment and Metabolic Syndrome: Molecular Basis

Epidemiological, clinical, and experimental evidence provides a solid basis for the hypothesis that IR is the pathophysiological origin for dyslipidemias, high blood pressure, and disorders in glucose homeostasis [30]. As studies of MS continued, a relationship emerged between obesity, the syndrome’s most prevalent individual criterion, and neurological alterations [31]. This association necessitates additional consideration of the pathophysiological mechanisms involved and how they are interconnected.

### 3.1. From Metabolic Syndrome to Cognitive Impairment

Epidemiological [32], neuroimaging [33], and animal modeling studies are available to characterize MS pathophysiology, accompanying diseases, and individual components in the development of neurodegenerative diseases and associated cognitive impairment [34]. The connection between diabetes mellitus (DM) and AD and the connection between obesity and cognitive impairment are two areas that have been investigated in depth, including reports of statistically significant relationships [10,35,36].

The hippocampus plays an important role in learning and memory. The effect of IR on hippocampal function has been widely studied [37]. Lindqvist et al. administered high- and low-fat diets to different groups of rats, using bromodeoxyuridine (BrdU) to observe synaptogenesis after 4 weeks. Male rats fed the high-fat diet showed a significant decrease in neurogenesis in the hippocampus. The animals did not develop obesity [38]. This result could reflect the role of lipid alteration, a component of MS, in cognitive decline.

Karimi et al. studied long-term neuronal potentiation (LTP) in the dentate gyrus (DG) of the hippocampus in mice receiving different combinations of a high-fat diet and antioxidants. Mice fed a high-fat diet showed lower LTP levels compared with control animals. In contrast, mice that received antioxidants displayed elevated LTP [39]. These effects might be mediated by an increase in free radical production caused by the high-fat diet, leading to oxidative stress (OS). This hypothesis is supported by rescue of LTP levels through the administration of antioxidants [39].

#### 3.1.1. Role of IR in the Formation of Amyloid-Beta Plaques

**Figure 1:**
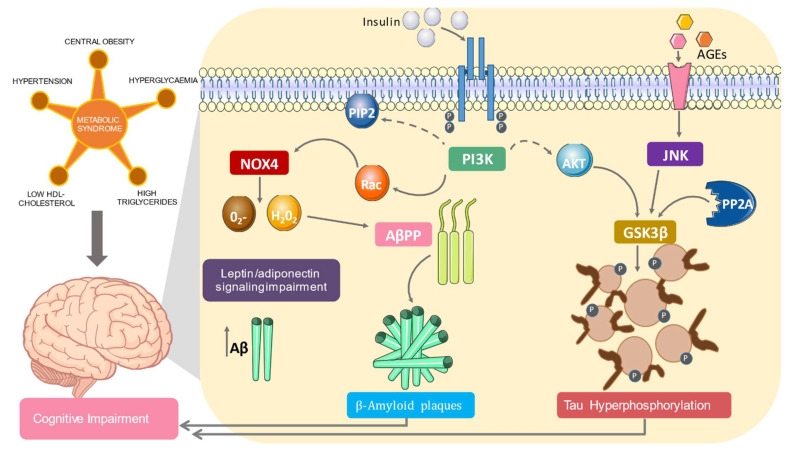
Impact of metabolic syndrome on cognitive impairment. PIP2: phosphatidylinositol bisphosphate; AKT: protein kinase B; PI3K: phosphoinositide kinase-3; JNK: c-Jun N-terminal kinase; AGEs: advanced glycation end products; PP2A: protein phosphatase 2A; GSK3β: glycogen synthase kinase 3 beta; NOX4: NADPH oxidase 4; 02-: superoxide; H_2_0_2_: hydrogen peroxide; Aβ: amyloid beta. Solid lines mean activation; dashed lines mean inactivation.

Production of reactive oxygen species (ROS) results in increased levels of amyloid precursor peptide-β (AβPP) and increased expression and accumulation of amyloid-β 42 (Aβ) [40]. This accumulation leads to the formation of amyloid-beta plaques, identified as a key element of AD [41]. One pathway for increased ROS production is increased insulin levels that lead to changes in normal NADPH oxidase (NOX4) pathway function. Elevated insulin levels induce phosphoinositol-3 kinase (PI3K) to phosphorylate Rac instead of phosphatidylinositol bisphosphate (PIP2). This alteration increases NOX4 activity and ROS levels. This aberrant metabolism is perpetuated because elevated ROS leads to activation of casein kinase 2 (CK2) and consequent activation of the retromer. This action signals the degradation of glucose receptor, GLUT4, leading to a continued increase in glucose levels in the blood and, therefore, increased production of insulin [42] (Figure 1).

The metabolic syndrome (MS) and its components cause brain alterations such as neuroinflammation, hyperphosphorylation of Tau, formation of beta amyloid plaques, and vascular changes (not represented in the figure). This is achieved through changes in the signaling of hormones such as adiponectin, leptin, and insulin. These changes are clinically expressed as mild cognitive impairment (MCI), Alzheimer’s disease (AD), and major vascular neurocognitive disorder.

This positive feedback produces an environment of neuronal inflammation, reported as a risk factor for AD. An associated hypothesis is that, along with neurofibrillary tangles and amyloid-beta plaques, inflammation has a critical role in the pathophysiology of the disease [43]. Insulin seems to play a significant role in the development of plaques, and hyperinsulinemia observed in IR leads to their formation. In vitro studies show that high levels of insulin affect the degradation and elimination of Aβ. Both insulin and Aβ are degraded by insulin-degrading enzyme (IDE). During hyperinsulinemia, IDE degrades insulin preferably to Aβ, promoting its oligomerization into insoluble aggregates [44]. In vivo experiments in rats corroborate these findings, showing that elimination of Aβ is reduced in the presence of high levels of insulin [45].

Insulin receptors provide an alternative explanation for IR effects on the hippocampus and other brain structures [46]. These receptors are abundant in metabolic active brain areas and exert their effects at the neuronal level via PI3K and mitogen-activated protein kinases (MAPK) pathways [47]. These pathways, when activated by insulin, promote angiogenesis in the brain. In the presence of IR, these pathways are not activated. This disruption might underlie the concomitant synaptic anomalies, memory disorders, decreases in neurogenesis at the hippocampus level, alterations in cognition, and decreases in levels of brain-derived neurotrophic factor (BDNF) [46].

#### 3.1.2. Metabolic Syndrome, Insulin Resistance, and Tau Proteins

Conversely, tau protein (TP) helps stabilize microtubules and its alteration results in the formation of neurofibrillary tangles [48]. Further, IR induces hyperphosphorylation of TP and induces cognitive impairment in human and animal models [49,50]. Thus, IR is associated with poorer performance on cognitive tests and higher levels of phosphorylated PT in cerebrospinal fluid (CSF) in cognitively normal individuals and carriers of the APOE allele ε4 [10,51].

One mechanism underlying this phenomenon involves glycogen synthase kinase-3β (GSK3β), a tau kinase regulated by insulin via the protein kinase B (AKT) pathway. Decreased brain insulin signaling caused by IR induces chronic exposure of neurons to high levels of insulin or an eventual decrease in insulin levels, resulting in PI3K dysfunction and reduced AKT-dependent phosphorylation. Downstream, GSK3β is activated, and ultimately TP is hyperphosphorylated [52,53]. The production of advanced glycation end products (AGEs) from OS damage via GSK3β receptors (RAGE) also increases the activity of GSK3β by an alternate pathway involving c-Jun N-terminal kinase (JNK) [54]. 

A recent study showed protein kinase A (PKA) is a potent tau kinase and its activation increases TP hyperphosphorylation in an insulin-deficient animal model [55]. Moreover, insulin deficiency influences hyperphosphorylated TP level by decreasing the activity of protein phosphatase 2 (PP2A) [56]. PP2A is the primary tau phosphatase involved in AD and its deregulation is associated with TP hyperphosphorylation [57]. Similarly, hypothermia, common in chronic DM, also leads to inhibition of PP2A activity [58]. 

Another pathological mechanism in AD is truncation of TP by proteolytic enzymes, such as caspases, peptidases, and thrombins that promote tau aggregation and formation of the central component of neurofibrillary tangles (NFT) [59]. DM stimulates apoptosis through the activation of caspases in affected tissues. Through hyperglycemia, DM might increase tau aggregation by activating caspases, thus contributing to AD risk [60]. Kim et al. demonstrated such increased tau aggregation in the brain of db/db rats using in vivo and in vitro type 2 diabetes mellitus (T2DM) animal models [61].

#### 3.1.3. Metabolic Syndrome, Leptin, Adiponectin, and Cognitive Disorders

Alterations in hormones involved in MS, such as leptin and adiponectin, are also linked to cognitive impairment [62]. Both hormones affect the metabolism of fatty acids and glucose as well as energy metabolism and food intake [63,64]. Their function in neuroplasticity, learning, and cognition [65,66] is now known via reports of leptin and adiponectin receptor expression in brain regions such as the hippocampus and neocortex [67].

Recent studies in animal models show that leptin deficiency or resistance is associated with cognitive disorders, such as reductions in LTP, long-term neuronal depression (LTD), and alterations in spatial memory [68]. Further, leptin modulates the production and elimination of Aβ in AD by inhibiting the formation of AβPP and increasing APOEε4-induced amyloid filament elimination. This activity may be mediated through the activation of AMP-activated protein kinase (AMPK) [69,70]. Leptin resistance in AD is associated with diminished activity in these pathways and increased cognitive impairment [71].

Additionally, adult rats deprived of adiponectin display several common characteristics of AD, including deposition of Aβ, TP phosphorylation, and neuroinflammation [72]. This observation is corroborated by Kim et al., who demonstrated that adiponectin receptor suppression also produced an AD-like phenotype [73] Thus, hormone deficiencies might be involved in AD pathogenesis. However, studies in humans are controversial since available information for the association of adiponectin and leptin levels in the blood and CSF with cognitive impairment is inconclusive [62].

#### 3.1.4. Metabolic Syndrome, Microvasculature, and Cognitive Impairment

Micro- and macrovascular changes observed in MS, such as hypertension and DM, are also associated with brain alterations, such as vascular neurocognitive disorder. However, several recent studies note the contribution of vascular risk factors in AD. Mechanisms for this accelerating cognitive decline are not fully elucidated [74]. 

Hypertension leads to alterations observed in magnetic resonance imaging (MRI), such as white matter lesions (WML), lacunar infarcts, microhemorrhages, and microinfarcts. All these abnormalities are part of a spectrum called small vessel cerebral disease (SVD), which is common in AD [75]. SVD is characterized by loss of smooth muscle cells in the mid-tunic, deposition of fibro-hyaline material, reduced light, and thickening of vascular walls [76]. Moss et al. studied Rhesus monkeys using an aortic coarctation model. Multiple microinfarcts and lesions in gray and white matter in hypertensive monkeys were associated with cognitive impairment [77]. Other mechanisms might involve large arteries via endothelial dysfunction that progresses to the formation of atherosclerotic plaques in the carotid or intracranial arteries. Such damage can cause ischemic events in brain regions related to cognition [78]. 

### 3.2. Exploring the Inverse Relationship—From Cognitive Impairment to Metabolic Syndrome

The consequences of MS on the development of cognitive impairment have been studied in depth [79], but the inverse relationship in which pathophysiological mechanisms of AD, such as hyperphosphorylation of TP and the formation of amyloid complexes-β, lead to the appearance of MS is largely unstudied.

#### 3.2.1. Tau Proteins and Deficits in Insulin Signaling

Interestingly, TPs, in addition to microtubule stabilization, also interact with insulin signaling pathway components in the brain. The N-terminal portion of TP can bind to homology 3 (SH3) domains of the Src family of tyrosine kinases, including domains of the p85 alpha subunit of PI3K, a key protein in the insulin signaling pathway. Under pathological conditions, hyperphosphorylation of TPs can lead to loss of functionality, triggering alterations in insulin signaling that eventually generate altered fasting glycemia and DM. The ability of TPs to interact with SH3 domains is inversely correlated with the degree of phosphorylation, suggesting that scaffolding properties of TPs are regulated by their phosphorylation status [80]. 

Further, co-immunoprecipitation studies of mouse brain tissue and N1E115 cells indicate that TPs bind to phosphatase and tensin homologous protein (PTEN), a negative insulin signal translocation regulator that catalyzes dephosphorylation of phosphatidylinositol triphosphate (PIP3) to PIP2. Thus, TP, by interacting with and inhibiting PTEN, promotes insulin signaling [81]. These studies raise the possibility that insulin helps maintain adequate brain activity due to TP and, conversely, pathological forms of TP could be harmful due to a loss of protein function. This suggestion is supported by a study that showed that TP removal was accompanied by loss of inhibitory effects of insulin on PTEN in the hippocampus, resulting in brain IR.

Concurrently, the absence of TP reduced the anorexigenic effect of insulin in the hypothalamus after intracerebroventricular injection of TP [81]. Previously, such injection induced increased food intake, weight gain, adiposity, hyperinsulinemia, and glucose intolerance in rodents with insulin receptor deletion in the hypothalamus [82,83]. These effects produce alterations in energy metabolism that may increase the risk of suffering from obesity, DM, and MS.

TP is also highly expressed in pancreatic islet β-cells. However, its function in peripheral tissues is not fully understood [84,85]. Wijesekara et al. investigated TP actions on β-cell function and glucose homeostasis using a tau KO rat model. Rats showed weight gain, defects in glucose signaling, and IR, leading to DM and ultimately MS. Thus, TP might be crucial for normal energy metabolism in peripheral tissues [86].

#### 3.2.2. Amyloid β and Insulinemic Alterations

In vitro and in vivo studies suggest that Aβ may also contribute to IR through various mechanisms. Aβ competitively inhibits the binding of insulin to its receptor [87] and activates the JAK2/STAT3/SOCS-1 signaling pathway to produce IR in the liver [88]. Further, the oligomer Aβ (AβO), a highly toxic species of Aβ, causes deregulation of N-methyl-D-aspartate (NMDA) receptors and leads to the production of excessive ROS. This effect is probably due to mitochondrial dysfunction [89]. 

This deregulation might lead to alterations in insulin signaling, since increased ROS activates several serine kinases, such as an inhibitor of the nuclear factor kappa-B kinase beta subunit (IKK-β), protein kinase C (PKC), and JNK. These kinases increase phosphorylation in Ser IRS-1 residues. ROS can cause OS and damage at mitochondrial and cellular levels. This stress generates mitophagy and, at high levels of stress, apoptosis. The elimination of mitochondria by mitophagy results in a decrease in oxidation and consequent accumulation of lipids, leading to IR and T2DM [90].

Conversely, AβO causes a rapid and substantial loss of insulin receptors in dendrites and inhibition of insulin receptor autophosphorylation associated with NMDA activity [91]. Additionally, an increase in levels of IR markers p(Ser)-IRS-1 and p-JNK were observed in neurons after intracerebroventricular injection of AβO in vivo in monkeys [92].

#### 3.2.3. Amyloid β, Tau Protein, and Leptin

Aβ and TP have also been linked to alterations in leptin signaling. Bonda et al. showed that TP hyperphosphorylation leads to the formation of NFT and dysfunction in intracellular trafficking networks in the hippocampus. Thus, the leptin receptor in its long form (Ob-Rb) becomes unable to reach cell membranes, hindering its access to circulating free leptin and interrupting signaling. This activity might lead to increased food intake and weight gain with subsequent development of obesity and long-term MS [93]; leptin in the hippocampus is associated with regulating food intake and processing food-related memories [94].

Elevated levels of Aβ1-42 produced by beta-site amyloid cleaving enzyme 1 (BACE1) increase leptin resistance in the hypothalamus, which is associated with decreased sensitivity to exogenous leptin throughout the body and exacerbation of body weight gain in rats fed high-fat diets. Thus, countering BACE1 activity may be protective against metabolic disorders [95].

The above findings affirm cognitive impairment as a key trigger of alterations in insulin signaling in the hypothalamus. The latter region is the primary regulator of body weight via controlling food intake and peripheral metabolism [96]. Thus, cognitive impairment might lead to metabolic changes that precede the development of MS and its complications.

## 4. Mild Cognitive Impairment and Metabolic Syndrome: Epidemiological Basis

Evidence concerning the relationship of MS and its components with MCI has accumulated in the last few years to the point where grouping these disorders into a single clinical entity, the cognitive–metabolic syndrome, may be appropriate [97]. Below, we summarize clinical and epidemiological information on the MCI and MS relationship and its components.

### 4.1. From Metabolic Syndrome to Cognitive Impairment

Cardio-metabolic risk factors and MS affect cognition and increase the risk of major neurocognitive disorders [98,99,100]. Speed of processing, attention, and executive functions are the most frequently affected domains [101,102]. Thus, an association is often reported between risk factors, such as hyperlipidemia, T2DM, obesity, hypertension, and physical inactivity, and models of risks of cardiovascular disease (CVD) (e.g., Framingham Risk Score) with the risk of MCI and major neurocognitive disorders (Table 1) [103,104,105,106]. 

Strong evidence of a link between high blood pressure in middle age and poorer cognitive function in old age is available [107,108]. Different prospective studies in older people show that increased blood pressure is associated with worse cognitive function [109]. The risk of cognitive impairment can increase up to 2.8 times [110]. In older women, risks may increase by up to 20% [111]. Similar results were reported in individuals from Hispanic [112], Swedish [113], Asian [114], and North American communities [115,116].

Obesity, defined by a high abdominal circumference or a body mass index (BMI) ≥ 30, is also associated with poor cognitive function [117,118]. Individuals with high BMI during middle age show low scores among various cognitive tests [119]. Further, long-term obesity is linked to lower cognitive performance and an increased risk of neurocognitive impairment in older people [120,121,122].

Several epidemiological studies and meta-analyses provide evidence for an effect of hyperlipidemia, hypertriacylglycerolemia, and HDL-C levels on cognitive performance in individuals with and without major neurocognitive disorders. Elevated LDL-C levels are correlated with the degree of cognitive impairment [123] and decreased episodic memory (ECM) [124]. Further, hypertriacylglycerolemia is associated with low scores in verbal tests [124,125]. Low concentrations of this lipoprotein are associated with poor and decreased memory in middle-aged adults [126], while in older people, low levels are associated with major neurocognitive disorders [127]. In contrast, improvement in cognitive test performance is reported for subjects over 75 years old with high HDL-C [128,129], which is also associated with a significant decrease in the appearance of major neurocognitive disorders [130].

Hyperinsulinemia, glucose intolerance, and T2DM are other cardio-metabolic risk factors that recently have been associated with cognitive impairment and different major neurocognitive disorders [131]. Hyperinsulinemia and impaired glucose tolerance, both indicators of a prediabetic state and an increased risk of developing DM, are associated with cognitive dysfunction and an increased risk of developing MCI [132,133,134,135]. These premorbid states are associated with reduced long-term memory scores [136] and impaired verbal fluency [137]. Lower performance on psychomotor and memory tests is observed in diabetic individuals [138]. These lower scores correlate with an increased risk of developing cognitive impairment and MCI [139,140,141,142,143]. 

Several studies associate different elements of MS with cognitive functions. However, few studies of MS as a clinical entity and its relationship with MCI or its progression to major neurocognitive disorders are available. Roberts et al. reported a cross-sectional study in 1969 of 70 89-year-old individuals. Participants with MS showed non-amnestic MCI (naMCI) when accompanied by elevated C-reactive protein (CRP). The combination of inflammation and MS might be linked to specific subtypes of MCI [144]. Yaffe et al. conducted a longitudinal, multicenter study with 4895 women with an average age of 66.2 years. MS was associated with an increased risk of developing cognitive impairment in older women. Risk increased by an age-adjusted 23% for each increment in the number of MS components [145]. Similar findings were reported by Pal et al. [146] and Atti et al. [147], who concluded that MS is associated with an increased incidence of major neurocognitive disorders and an increased risk of progression from MCI to such disorders, respectively.

**Table 1:** Effects of components of MS on cognitive function, the risk of MCI, and major neurocognitive disorders.

MS Component	Authors (REF)	Methodology	Results
High blood pressure	McDonald et al. [109]	Longitudinal cohort study of the association between cognitive function and BP variability in adults ≥65 years.	After 5 years of monitoring, diurnal systolic BP variability was independently associated with a greater decrease in total CAMCOG (CV: 3.205; *p* = 0.043) and MMSE (CV: 3.985; *p* = 0.020) scores.
	Haring et al. [111]	Prospective study in 6426 cognitively intact older women of the relationship between hypertension and cognitive impairment.	Hypertension was associated with an increased risk for cognitive decline (HR: 1.20; 95% CI: 1.04–1.39; *p* = 0.02).
Obesity	Sabia et al. [121]	Longitudinal cohort study of the association between BMI and mid-age cognition throughout adult life in 5131 individuals.	Late midlife obesity was associated with lower scores on the MMSE and on memory and executive function scores compared with normal-weight individuals (mean difference (95% CI): −0.99 (−1.78–0.21), −0.82 (−1.57–0.08), and −0.80 (−1.49–0.12), respectively (*p* < 0.05)).
	Beydoun et al. [122]	Meta-analysis of the association between obesity and major neurocognitive disorder in older adults.	A significant U-shaped association was found between BMI and major neurocognitive disorder (*p* = 0.034), with an increased risk of disorder (OR (95% CI): 1.42 (0.93–2.18)) and increased incidence of AD (OR (95% CI): 1.80 (1.00–3.29)) in obese individuals.
Dyslipidemias	de Frias et al. [124]	Longitudinal cohort study of the association between total cholesterol, triglycerides, and cognitive performance in older adults.	Hypertriglyceridemia was associated with a low score on the verbal memory tests (γ = −2.31; *p* < 0.05), while hypercholesterolemia was associated with a detrimental effect on facial recognition test score (γ = −2.13; *p* < 0.05).
	Singh-Manoux et al. [126]	Longitudinal cohort study of the relationship between HDL-c and verbal short-term memory in middle-aged adults.	After 5 years of monitoring, decreased HDL-C was associated with decreased verbal memory (OR = 1.61; 95% CI = 1.19–2.16).
DM	Elias et al. [117]	Longitudinal study of the effects of T2DM on cognitive performance of adult individuals.	The amount of time suffering from diabetes was associated with poorer cognitive performance (β = −0.02; *p* < 0.02).
	Kanaya et al. [137]	Longitudinal cohort study of changes in cognitive performance according to glucose tolerance status in older adults.	After 4 years, women with DM had a 4-fold increased risk of cognitive impairment (OR (95% CI): 4.38 (1.71–11.27); *p* = 0.02).
MS	Atti et al. [147]	Meta-analysis of the relationship between MS and progression to major neurocognitive disorder in individuals with MCI.	Having MS increased the risk of progression from MCI to major neurocognitive disorder (HR (95% CI): 2.69 (1.16–6.27); *p* < 0.05).
	Pal et al. [146]	Meta-analysis that quantified the relative risk of progression from MCI to major neurocognitive disorder in individuals with MS.	An increased risk of progression was found in individuals with MCI and SM (OR (95% CI): 2.95 (1.23–7.05) *p* < 0.05).

Abbreviations: MS: metabolic syndrome; MCI: mild cognitive impairment; BP: blood pressure; CAMCOG: Cambridge Cognitive Examination; MMSE: Mini-Mental State Examination; CV: coefficient of variation; BMI: body mass index; OR: odds ratio; HR: hazard ratio; CI: confidence interval; AD: Alzheimer’s disease; HDL-c: high-density lipoprotein; DM: diabetes mellitus.

The impact of MS on cognitive function is not limited to adults. There is also evidence that suggests that MS components may be detrimental in younger populations. The presence of T2DM, obesity, and hypertension in children and adolescents is associated with poorer performance in overall functioning, and declines in executive function, memory, attention, and intelligence quotient (IQ) [148,149,150,151,152].

Cardiovascular and metabolic risk factors are modifiable and their timely identification and consequent management could prevent MCI or its progression to major neurocognitive disorders [101]. Thus, lifestyle changes, including increased physical activity and implementation of healthy diets, and antihypertensive, hypolipidemic, and insulin-sensitizing drugs are important considerations for the management of premorbid state characteristics of MS (Table 2) [153].

Changes in lifestyle and physical activity positively impact cognitive function. [154,155]. Physical activity is associated with better scores on tests of executive function, processing speed, and improvement in global cognitive function. These benefits were found both in healthy older subjects and in older subjects with MCI or major neurocognitive disorders [156,157,158,159]. More studies to elucidate types of exercise, times, and intensity needed to cause a positive impact on cognition are necessary; still, 150 min of physical activity per week is proposed to improve the brain health of individuals with MCI [160]. 

Better results are obtained if physical activity is combined with a healthy diet. Supplementation with B-vitamins, folic acid, docosahexaenoic acid (DHA), eicosapentaenoic acid (EPA), and flavonoids is associated with improved cognitive performance, particularly memory, in subjects with MCI [161]. Similarly, both cognitively normal individuals and those with MCI are reported to be at less risk of developing MCI or AD if they maintain high adherence to a Mediterranean diet [162]. Similar results are associated with Mediterranean-DASH diets [163], low-carbohydrate diets (keto-diet) [164], and fish PUFA diets [165]. 

Additionally, a causal relationship between antihypertensive drugs and improved cognitive function is supported by available evidence. Antihypertensive drugs, especially calcium channel blockers and renin–angiotensin system blockers, have a protective effect on cognitive decline and decrease the risk of AD and neurocognitive vascular disorders in older people [166]. Similarly, treatment with antihypertensive drugs reduces the risk of major neurocognitive disorders by 9% and shows improvement in all cognitive domains, except language [167]. Longitudinal studies that included older individuals without major neurocognitive disorders who were undergoing antihypertensive therapy produced supporting results [110,168].

Controlling glycemic concentrations and increasing peripheral insulin sensitivity are strategies that might positively affect cognitive function [146]. A recent meta-analysis showed that treatment with metformin or sulfonylureas is associated with a significant decrease in cognitive impairment in patients with T2DM. In contrast, the use of insulin aggravated the dysfunction [169]. Studies of metformin as monotherapy [170], or combined with vildagliptin [171], on the participants’ cognitive function produced similar results. However, other studies show no association between the use of antidiabetic drugs and improvement in cognitive function [172,173]. One study linked the use of such drugs to the diagnosis of MCI [174]. 

Finally, unlike antihypertensive and antidiabetic drugs, hypolipidemics, such as statins, do not affect the risk of progression to MCI or major neurocognitive disorders of any kind [175,176]. Indeed, several clinical and epidemiological studies report no significant association between statin use and reduced cognitive impairment [177,178,179,180].

**Table 2:** Association between treatment of MS elements and MCI improvement.

Therapeutic Approach	Authors (REF)	Methodology	Results
Lifestyle changes	Karssemeijer et al. [157]	Meta-analysis of the effect of cognitive and physical exercise intervention in older adults with MCI or major neurocognitive disorder.	A positive effect of the combination of physical–cognitive interventions on global cognitive function was observed (MDS (95% CI) = 0.32 (0.17–0.47); *p* < 0.05). It was equally beneficial for individuals with MCI (MDS = 0.39 (0.15–0.63); *p* < 0.05), and for patients with major neurocognitive disorder (MDS = 0.36 (0.12–0.60); *p* < 0.001).
	Zhang et al. [165]	Meta-analysis of the association between risk of cognitive impairment and intake of fish and PUFAs.	Increased fish consumption was associated with decreased risk of major neurocognitive disorder (RR: 0.95; 95% CI: 0.90–0.99; *p* = 0.042). A significant curvilinear relationship was observed between PUFA consumption and MCI risk (*p* nonlinearity < 0.001).
	Krikorian et al. [164]	Prospective study of the effect of a ketogenic diet in older individuals with MCI.	An improvement in verbal memory performance was observed in patients on a low-carbohydrate diet (*p* = 0.001). Memory performance was positively correlated with ketone levels (*p* = 0.04).
Antihypertensives	Tzourio et al. [110]	A longitudinal study of the effect of antihypertensive drugs on the risk of cognitive decline in older individuals.	The risk of cognitive impairment was higher in untreated subjects (OR = 6.0 (95% CI: 2.4–15.0)), compared with subjects treated with antihypertensives (OR = 1.3 (95% CI: 0.3–4.9)).
	Guo et al. [168]	Prospective study that evaluated whether the use of antihypertensives affected the appearance and progression of major neurocognitive disorders in older adults.	The risk of major neurocognitive disorder was reduced in subjects receiving antihypertensive treatment and without major neurocognitive disorder at the beginning of the study (RR = 0.7 (95% CI: 0.6–1.0); *p* = 0.03).
Antidiabetics	Ng et al. [170]	Longitudinal study of the protective effect of metformin on the cognitive performance of older adults.	Use of metformin showed an inverse association with cognitive impairment (OR = 0.49 (CI 95%: 0.25–0.95); *p* < 0.05), and was associated with a low risk of cognitive impairment after 6 years of use (OR = 0.27 (CI 95%: 0.12–0.60); *p* < 0.05).
	Borzì et al. [171]	Retrospective study of the effect of vildagliptin on cognitive function in older diabetic adults with MCI.	The use of metformin as monotherapy or in combination with vildagliptin was associated with a significant reduction in MMSE score (*p* < 0.001).
Hypolipidemics	Bosch et al. [177]	Clinical trial on the effect of rosuvastatin in reducing cognitive impairment in older adults.	The mean difference in DSST score between rosuvastatin vs. placebo was −0.54 (95% CI: −1.88–0.80); *p* < 0.05.
	Bettermann et al. [178]	Clinical trial on the impact of statin use on delaying cognitive decline in patients with and without MCI.	Statins were associated with a decreased risk for increased neurocognitive impairment from all causes in patients who did not have MCI at the beginning of the study (HR = 0.79 (95% CI: 0.65–0.96) *p* = 0.021). In subjects with MCI, these protective effects were not observed.

Abbreviations: MS: metabolic syndrome; MCI: mild cognitive impairment; SMD: standardized mean difference scores; PUFAs: polyunsaturated fatty acids; MMSE: Mini-Mental State Examination; CV: coefficient of variation; OR: odds ratio; RR: relative risk; HR: hazard ratio; CI: confidence interval; DSST: Digit Symbol Substitution Test.

### 4.2. Exploring the Reverse Relationship—From Cognitive Disorder to Metabolic Syndrome

Epidemiological studies suggest the inverse relationship. MCI has been assessed as a contributor to the development of MS (Table 3). In a cross-sectional study of 3312 male and female participants aged 70 years and older in Japan, a higher prevalence of MS was observed in subjects with naMCI than in those with normal cognition. Moreover, women with naMCI had high blood pressure and high glucose levels more often, while men with naMCI showed only a higher frequency of high glucose levels compared with the control group. However, a causal relationship between the two could not be determined from this cross-sectional study [181]. Clinical evidence is still scarce and has focused more on specific components of MS, such as insulin/glucose alterations and T2DM, than on MS as an entity.

Alterations in insulin signaling have been reported in postmortem studies in brains from individuals with AD [182,183], as well as in patients with AD in clinical studies of plasma hyperinsulinemia and reductions in insulin levels in the CSF. These changes worsen as the disease progresses [184]. Animal models produce similar results [88,185]. Accordingly, Janson et al. used the Mayo Clinic Alzheimer’s Disease Patient Registry to show a higher incidence of both T2DM and IR in 80% of AD patients. A greater increase in fasting plasma glucose (FPG) with age compared with the control group was also observed. AD patients might thus be at greater risk of developing a diabetic phenotype and suffering from T2DM [186]. 

Similarly, a longitudinal study using data from the Lothian Birth Cohort of 1936 (LBC1936) examined parameters, such as cognitive changes and glucose levels. This cohort consists of 1091 initially healthy individuals born in 1936. Individuals were assessed using glycosylated hemoglobin (HbA1c) data for four ages—70, 73, 76, and 79 years. Lower cognitive function at 70 years was associated with increased HbA1c in the following decade. Cognitive dysfunction is thus negatively correlated with increases in HbA1c. Maintaining high cognitive function could be a protective factor for the development of hyperglycemia and T2DM [187]. 

Likewise, Peng et al. initially conducted a cross-sectional study in 2126 participants, including 1063 patients recently diagnosed with T2DM and 1063 patients with standard glucose tolerance. Individuals with higher plasma concentrations of both Aβ40 and Aβ42 were more likely to have T2DM compared to subjects with the lowest concentrations [188]. In a follow-up study, the authors examined Tongii-Ezhou Cohort (TJEZ) data prospectively. One hundred and twenty-one individuals with T2DM and 242 healthy individuals were included. The same association was found, where the probability of T2DM was higher with higher plasma concentrations of Aβ, 3.79 (95% CI 1.81–7.94) for Aβ40 and 2.88 (95% CI 1.44–5.75) for Aβ42. The authors conclude that a positive association exists between Aβ and the risk of acquiring T2DM [188].

**Table 3:** Effect of cognitive dysfunction and suffering from MS or MS components.

Author (REF)	Methodology	Results
Janson et al. [186]	Longitudinal study where prevalence of T2DM in patients with AD was evaluated, along with the association between FPG and aging in these patients.	The prevalence of T2DM (34.6 vs. 18.1%; *p* < 0.05) and IFG (46.2 vs. 23.8%; *p* < 0.01) was higher in the AD group vs. the control group. A greater increase was seen in FPG per year in the AD group (0.83 vs. 0.57 mg/dL^−1^; *p* < 0.01).
Bae et al. [181]	Cross-sectional study of the prevalence of MS by type of MCI in 3312 older adults and differences related to sex.	The prevalence of MS was higher in participants with naMCI (men: *p* = 0.030; women: *p* = 0.040) and the risk of MS was higher in men (OR = 2.45; 95% CI: 1.13–5.32) than in women (OR = 1.94; 95% CI: 1.12–3.39) compared with participants with normal cognition.
Altschul et al. [187]	Longitudinal cohort study of the association between cognitive function, HbA1c, and other variables in early and late life in 1091 adults.	High cognitive function at age 11 predicted low HbA1c levels at age 70 (*p* < 0.001). Additionally, high cognitive function at age 70 was associated with a smaller increase in HbA1c levels between age 70 and 79 (*p* < 0.001).
Peng et al. [188].	Study comparing 1063 newly T2DM diagnosed individuals with 1063 control individuals for an association between plasma concentrations of Aβ40 and Aβ42 with risk of T2DM.	The risk of T2DM was higher in individuals with the highest concentrations of Aβ40 and Aβ42 (OR = 2.96 (95% CI: 2.06–4.25)) compared with subjects with the lowest concentrations of Aβ.
Peng et al. [188].	Prospective study of the association between plasma concentrations of Aβ40 and Aβ42 with risk of T2DM.	A higher risk of T2DM was found in individuals with concentrations greater than that of Aβ (OR = 3.79 (95% CI: 1.81–7.94)) for Aβ40 and (OR = 2.88 (95% CI: 1.44–5.75)) for Aβ42.

Abbreviations: T2DM: diabetes mellitus type 2; HbA1c: glycated hemoglobin; FPG: fasting plasmatic glucose; IFG impaired fasting glucose; MCI: mild cognitive impairment; naMCI: non-amnestic mild cognitive impairment; Aβ: amyloid-beta; OR: odds ratio; CI: confidence interval.

These findings imply that therapeutic intervention aimed at MCI, especially AD, could be beneficial for treating MS and its components (Table 4). A drug approved for the treatment of moderate-to-severe AD is memantine, an NMDA receptor antagonist that reduces the accumulation of Aβ in AD patients [189]. Ettcheto et al. analyzed the effects of memantine in rats with model AD that were fed a high-fat diet. After 12 weeks of treatment with 30 mg/kg memantine, improvement of peripheral metabolic parameters, such as IR, was observed [190]. 

Similarly, Ahmed et al. investigated piracetam and memantine in the treatment of T2DM in 120 individuals. Piracetam is used to improve memory and brain function. Diabetic patients with AD treated with either drug showed a significant reduction in diabetic markers (GPA, HbA1c%, and insulin levels) compared to a symptomatic control group. Thus, agents used to treat MCI demonstrate a therapeutic potential for the treatment of metabolic disorder [191].

Another therapeutic strategy is based on reducing the activity of enzymes that promote the formation of Aβ, such as BACE1. The metabolic role of BACE1 is not fully understood, though loss of BACE1 in transgenic rats leads to increased sensitivity to insulin and decreased body weight [192]. Its mechanisms of action may involve leptin signaling and thermogenesis [95]. These results were extrapolated in a randomized clinical trial (RCT). Patients with AD treated with lanabecestat, a BACE1 inhibitor, showed greater weight loss than a placebo group after 104 weeks of treatment [193]. 

Additionally, immunotherapy against Aβ is used to improve insulin sensitivity and plasma glucose levels. Zhang et al. used an APP/PS1 EA rat model with increased plasma levels of Aβ40/42. Animals exhibited altered glucose/insulin tolerance and liver insulin signaling. After nine months of intraperitoneal injections of antibodies against Aβ, an improvement was observed in insulin sensitivity. Hepatic signaling of JAK2/STAT3/SOCS-1 compared to the control group was concurrently attenuated. Thus, neutralization of Aβ attenuates hyperglycemia and IR in vivo [194].

**Table 4:** Summary of preclinical and clinical studies exploring treatment of SM with anti-Alzheimer’s drugs.

Author (REF)	Treatment	Methodology	Results
Ettcheto et al. [190]	Memantine	Preclinical study of the effects of MEM on learning and memory impairment in rats with familial AD and HFD-induced insulin resistance.	MEM prevented body weight increase in HFD-fed mice with APP/PS1 (*p* < 0.001). Hepatic IR protein levels showed a significant increase in APP/PS1 MEM mice compared to nontreated controls (*p* < 0.05), improving insulin function in the liver.
Zhang et al. [194]	Anti- Aβ Immunotherapy	Preclinical study of the effects of intraperitoneal injections of anti-Aβ antibodies in APP/PS1 rats on glucose metabolism.	After 9 months of treatment, neutralization of Aβ reduced fasting blood glucose level (*p* < 0.001), improved insulin sensitivity (*p* < 0.05), and inhibited hepatic JAK2/STAT3/SOCS1 signaling (*p* < 0.05) in APP/PS1 AD model rats.
Wessels et al. [193]	Lanabecestat	RCT that assessed whether lanabecestat slows the progression of AD compared with placebo in patients with early AD (mild cognitive impairment) and mild AD dementia.	Even though treatment with lanabecestat did not slow cognitive decline, patients who completed week 104 had a mean (SD) weight loss of 0 (4.7) kg for placebo, −0.8 (4.6) kg for patients treated with 20 mg lanabecestat, and −1.9 (5.2) kg for those treated with 50 mg.
Ahmed et al. [191]	MemantinePiracetam	Clinical study of the effect of piracetam and memantine on diabetes mellitus.	A significant decrease in all diabetic markers (FPG, HbA1c%, and insulin levels) in the diabetic and Alzheimer’s patients was observed after treatment with memantine or piracetam compared to diabetic and Alzheimer’s patients with symptomatic treatment (*p* < 0.05).

Abbreviations: MEM: memantin; AD: Alzheimer’s disease; HFD: high-fat diet; Aβ: amyloid-beta; RCT: randomized clinical trial; FPG: fasting plasmatic glucose; HbA1c: glycated hemoglobin.

Numerous epidemiological and clinical studies and meta-analyses provide evidence that MS and its components have a substantial impact on the development of MCI. However, the inverse relationship, where MCI contributes to MS risk, is feasible, though some studies report the lack of association between these clinical entities [195,196,197,198]. A causal relationship between MS and MCI has yet to be conclusively identified.

## 5. Conclusions

The current epidemic of metabolic disorders, framed in what we term MS, increases in a society with unhealthy lifestyles, and many aspects of this condition are still unknown. Research on MS generates a constant stream of new information and a flood of debate on whether this pathology exists, its components, and the pathophysiological mechanisms that produce it. 

Recently, a two-way relationship between MS and brain disorders, such as MCI and AD, has been observed, but without clarity regarding which phenomenon occurs first and which pathophysiological pathways are involved. Contrasting findings could be attributed to factors inherent in the complex nature of MS and MCI. Both disorders are multifactorial and display disparity in clinical manifestations. Further, research methodology is heterogeneous, reflecting the variability in criteria used to define MS, methods used for evaluating cognitive function, study design, and the presence of confounding factors. The latter factors might be varying characteristics of the populations studied, such as age, sex, race, educational status, socioeconomic status, and health–disease status. Large-scale studies with adequate power and longer follow-up periods will be necessary to establish a direct and accurate causal relationship between MS and MCI pathologies.
